# Evidence for arthrogenic inhibition of the gluteus medius after anterior cruciate ligament injury: A systematic review

**DOI:** 10.1002/jeo2.70551

**Published:** 2025-11-14

**Authors:** Jean Tarchichi, Jad Zalaket, Nicolas Graveleau, Pierre Laboudie, Nicolas Bouguennec

**Affiliations:** ^1^ Clinique du Sport, CCOS Mérignac France; ^2^ Department of orthopaedic surgery Hôtel‐Dieu de France Hospital Beirut Lebanon

**Keywords:** ACL, AMI, arthrogenic muscle inhibition, gluteus medius, weakness

## Abstract

**Purpose:**

Arthrogenic muscle inhibition (AMI) is a reflex inhibition following joint injury or surgery affecting periarticular muscles. While AMI has been extensively studied in the quadriceps after anterior cruciate ligament (ACL) injury, its potential impact on the gluteus medius remains unclear. Given the muscle's critical role in pelvic and lower limb control, clarifying this relationship may have important implications for rehabilitation strategies after ACL injury. The purpose of this study is to search for a relation between ACL injuries and arthrogenic inhibition of the Gluteus medius in the ipsilateral hip.

**Methods:**

A structured review of 12 peer‐reviewed studies was conducted, assessing Gluteus medius or hip muscle function after ACL injury or reconstruction. Eligible studies evaluated muscle activation, strength and neuromuscular adaptations using electromyography (EMG), dynamometry or biomechanical motion analysis. Data were summarised to evaluate the presence and clinical significance of AMI affecting the gluteus medius.

**Results:**

Among the included studies, two reported clear signs of gluteus medius inhibition after ACL injury or reconstruction. Several demonstrated reduced activation and weakness of the gluteus medius, suggesting a potential inhibitory mechanism. Specifically, EMG‐based findings in two studies supported altered neuromuscular recruitment patterns, while three others identified movement strategies with compensation or abductor weakness. However, some other studies reported no significant deficits, highlighting interstudy variability due to differing methods, population characteristics or time since injury. Despite these differences, a general trend towards proximal neuromuscular adaptation appears relevant.

**Conclusion:**

There is emerging evidence that suggests the presence of gluteus medius dysfunction suggestive of AMI following ACL injury in certain individuals. While not universally observed, this inhibition may impair dynamic hip stability and functional recovery. Future research should explore whether targeted hip muscle training can reduce persistent neuromuscular deficits after ACL injury.

**Level of Evidence:**

Level III.

## Graphical abstract

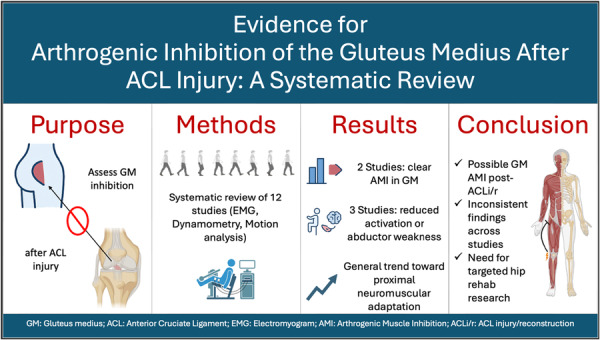

**Abbreviations**
ACL: anterior cruciate ligament
ACLi: anterior cruciate ligament injury
ACLr: anterior cruciate ligament reconstruction
AMI: arthrogenic muscle inhibition
AMR: arthrogenic muscle response
CNS: central nervous system
EMG: electromyogram
PFPS: patello‐femoral pain syndrome
PRISMA: preferred reporting items for systematic reviews and meta‐analyses
RCTs: randomised controlled trials

## INTRODUCTION

Arthrogenic muscle inhibition (AMI) is a known complication of anterior cruciate ligament injury/reconstruction (ACLi/r) especially for the quadriceps muscle [11, 30, 33, 34, 35]. This neurophysiological process (unlike generalised muscle weakness) is characterised by reduced ability to fully activate a muscle due to altered afferent input from the injured joint, which disrupts normal spinal reflexes and motor cortical drive. It compromises muscle strength, and eventually generates abnormal movement biomechanics [28]. Individuals who develop such muscular weakness and poor neuromuscular control of the injured limb are therefore at an elevated risk of re‐injury after their surgical reconstruction [10, 20, 22, 24, 42].

Being a primary hip abductor, the Gluteus medius maintains the stability of the pelvis during the stance phase of gait since it controls eccentrically femoral internal rotation [2, 3]. Any dysfunction in the Gluteus medius will therefore lead to contralateral pelvic drop or increased hip internal rotation, which amplifies the valgus force at the knee, a known risk factor for noncontact ACL injury [2, 14].

AMI of the hip muscles including the Gluteus medius has already been described in other pathologies affecting the ipsilateral knee and ankle. In a controlled laboratory study, Van Deun et al. observed delayed hip muscle activation onset times in patients with chronic ankle instability, while transitioning from double‐leg stance to single‐leg stance [38]. Furthermore, some studies highlighted the impaired function of the hip abductors in patients with patello‐femoral pain syndrome (PFPS) [1, 18, 19, 23, 41].

To this day, the potential deficits in the hip neuromuscular function that may be found after an ACL injury or reconstruction are not really considered, as these alterations remain unclear [7, 24, 37]. Most research of AMI to date focus on the Quadriceps [11, 15, 22, 34], and less attention is directed towards the Gluteus medius. Impairement of the latter has important clinical implications, including altered limbs biomechanics, compromised trunk and hip stability, and potentially increased risk of secondary injury. Enhancing our understanding of neuromuscular control deficits following ACL injury is therefore crucial. Therefore, the aim of this review was to search for a relation between ACL injuries and arthrogenic inhibition of the Gluteus medius in the ipsilateral hip.

## METHODS

This study was designed as a systematic review of the current literature according to the preferred reporting items for systematic reviews and meta‐analyses (PRISMA) guidelines [21].

### Search strategy

A search was conducted on the 10 March 2025 in the following databases: PubMed, Embase, Cinahl, Cochrane Central Register of Controlled trials and AusportMed. The search included the following keywords combined with an AND: (‘Arthrogenic’ OR ‘AMI’ OR ‘inhibition’ OR ‘Neuromuscular’ OR ‘Motor control’ OR ‘Muscle Weakness’) AND («Glut*» OR «Hip» OR «Trunk» OR «Medius» OR «Abdu*») AND (‘Knee’ OR «ACL» OR «Menisc*» OR ‘Patellofemoral’ OR ‘Knee osteoarthritis’ OR ‘total knee arthroplasty» OR «TKA»). This wide array of keywords was used to not miss any potential article that could be related to the concept of AMI following a knee pathology. Two additional filters were added to only include studies published in English and involving human subjects. Duplicates and irrelevant papers were removed judging by their title first, then by the abstract. Full‐text assessment of the remaining papers was then performed.

### Selection criteria

The following inclusion criteria were applied on the final yield:

− Published peer‐reviewed study: randomised controlled trials (RCTs), nonrandomised comparative studies and retrospective cohort studies.
− Functional outcome data of hip muscles assessed by a quantifiable method.
− Presence of an ACL injury, regardless of the treatment with reconstruction or simple physical therapy.
− Study in the English language.

All criteria had to be satisfied for a study to be included in the final review.

Exclusion criteria were as follows:

− Reports on guidelines, technical notes, reviews, systematic reviews and meta‐analysis.
− Studies that included other ligamentous injuries in the knee than the ACL such as medial collateral ligament, lateral collateral ligament, posterior cruciate ligament, postero‐lateral corner or postero‐medial corner injuries, to avoid confusion bias in final analysis.
− Studies that only concerned AMI in the knee muscles without involving hip muscles, as the main objective was focused on the Gluteus medius.
− Studies that only concerned AMI in the hip after PFPS or knee osteoarthritis, without an ACL injury.

After applying the selection criteria, the title and abstract of each study were reviewed. In the cases where it was not clear from the review of the title and abstract whether a study was appropriate for inclusion, the full text of the article was examined. Two reviewers applied the selection criteria independently. Consensus was used to resolve any disagreements between reviewers, with the senior author if consensus was not achieved.

**Figure 1:**
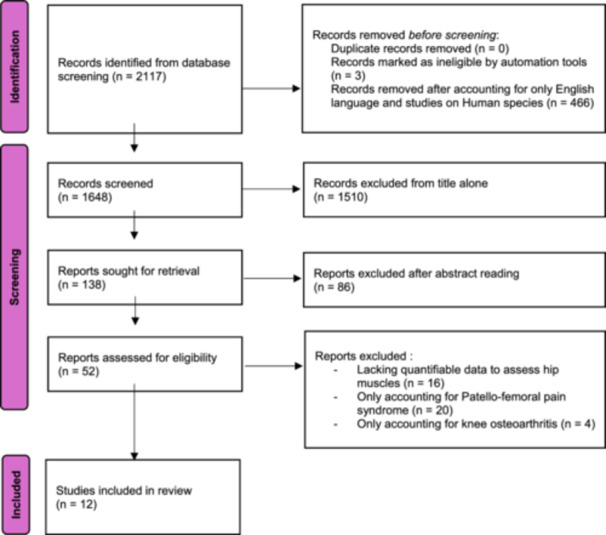
Preferred reporting items for systematic reviews and meta‐analyses (PRISMA) flow diagram.

A total of 2117 records were retrieved from the initial database search. After accounting for English language articles and articles involving only human species, 1651 articles were obtained and then uploaded to Zotero. No duplicates were identified. Three articles were then retracted for fake peer review and unreliable results. From the remaining 1648 papers, we narrowed it down to 138 articles based on the title alone. After reading their abstracts, 86 more articles were excluded. For the final 52 papers, 16 were excluded since they lacked a quantifiable data to assess hip muscles. Finally, out of the remaining 36 papers, 20 only accounted for patellofemoral pain syndrome, and 4 only involved knee osteoarthritis and total knee arthroplasty. Twelve articles reporting on hip muscle activity after and ACL injury were therefore included for the final qualitative synthesis after a scrupulous check of the full text (Figure 1).

### Data extraction

Two authors independently extracted the data, which were entered into a specifically designed spreadsheet containing headings for the selected outcomes. The following data was obtained from each paper: name of the first author, year of publication, study type, number of patients, the status of the ACL pathology (injured, reconstructed, or both), mean date of follow‐up, sex, age, height, body mass, type of graft used for ACLr if any, method of assessing AMI, hip muscle groups examined, tests performed, pre‐ and post‐op type of rehabilitation, and key findings.

### Best evidence synthesis

To assist with evaluating the outcome findings that could not be assessed through meta‐analysis due to the limited availability of homogenous data, a best evidence synthesis using RCTs was performed. The method proposed by Van Tulder et al. [39] and adapted by Steultjens et al. [17] was used to ascribe levels of evidence of effectiveness, taking into consideration study design, methodological quality and statistical significance of the findings (Supporting Information S1: Appendix 1).

## RESULTS

### Methodological quality

The methodological quality of the included papers was assessed with the modified Downs and Black score (Table 1), which is appropriate for cohort study designs and has previously been found to be reliable [8]. A modified version in this study was used, with a maximum score of 16; a total score ≥12 is thought to be high quality, 10 or 11 to be moderate quality, and ≤9 low quality [5]. The methodological quality of each article was stratified by the first author.

**Table 1:** Quality assessment tool: modified Devitt et al. and Downs and Black [5, 8].

	Flaxman et al. [9]	Noehren et al. [24]	Sharifmoradi et al. [31]	Nyland et al. [25]	Ortiz et al. [26]	Srinivasan et al. [36]	Rostami et al. [4]	Dalton et al. [3]	Thomas et al. [37]	Dingenen et al. [6]	Dingenen et al. [7]	Smeets et al. [32]
Is the hypothesis/aim/objective clearly described?	1	1	1	1	1	1	1	1	1	1	1	1
Are the main outcomes to be measured clearly described?	1	1	1	1	1	1	1	1	1	1	1	1
Are the characteristics of the patients clearly described?	1	1	1	1	1	1	1	1	1	1	1	1
Are the interventions of interest clearly described?	1	1	1	1	1	1	1	1	1	1	1	1
Are the distributions of principal confounders clearly described?	0	1	1	1	0	1	0	0	1	1	1	1
Are the main findings clearly described?	1	1	1	1	1	1	1	1	1	1	1	1
Does the study provide estimates of random variability in the data?	1	1	1	1	1	1	1	1	1	1	1	1
Have actual probability values been reported (e.g., 0.035 vs. <0.05)?	1	1	1	1	1	1	1	1	1	1	1	1
Were main outcome measures valid and reliable?	1	1	1	1	1	1	1	1	1	1	1	1
Were participants in different intervention groups recruited over the same time period?	1	1	1	1	1	1	1	1	1	1	1	1
Were study subjects randomised?	0	0	0	0	0	0	0	0	0	0	0	0
Were baseline characteristics of groups similar?	1	1	1	1	1	1	1	1	1	1	1	1
Were analyses adjusted for confounding variables?	0	1	1	1	0	1	0	0	1	0	0	1
Were losses to follow‐up taken into account?	0	0	0	1	0	0	0	1	0	0	0	0
Was there sufficient power to detect a clinically important effect?	0	1	1	0	1	1	1	0	1	1	1	1
Total	10	13	13	13	11	13	11	11	13	12	12	13
Quality	Moderate	High	High	High	Moderate	High	Moderate	Moderate	High	High	High	High

*Note*: Methodological quality of the articles as per the modified Downs and Black score. 1 point is accorded if the answer is ‘Yes’, and 0 point if the answer is ‘No’. A total score ≥12 = high quality, 10–11 = moderate quality, ≤9 = low quality.

The methodology quality scores ranged from 10 to 13 out of a maximum of 15 points. Eight studies were of high quality [6, 7, 24, 25, 31, 32, 36, 37], while the other four were of moderate quality [3, 4, 9, 26]. None of the studies had subjects randomised, and only two of them took into account losses to follow‐up [3, 25]. Only three studies didn't have sufficient power to detect a clinically important effect [3, 9, 25].

### Demographic characteristics

The 12 studies reported a total of 510 patients, 311 ACL pathologies (61%) and 199 healthy controls (39%). Out of those 311 ACLs, 223 had a reconstruction (72%) and 88 were only injured (28%). The population characteristics are listed in Table 2. Only one study did not mention the gender [31]. The procedures involved an ACLr with Hamstrings autograft (six studies), Bone‐tendon‐bone autograft (four studies), allografts (three studies) and patellar tendon (one study).

**Table 2:** Qualitative data.

Reference	Year of publication	Study type	Number of patients	Status of ACL (injured, reconstructed, both)	Mean date of follow‐up	Sex	Age (years)	Height (cms)	Body mass (kgs)	Type of graft for ACLr	Method of assessing AMI	Hip muscle groups examined	Tests performed	Type of rehabilitation (pre‐op)	Type of rehab (post‐op)	Key findings
Flaxman et al. [9]	2019	Case‐control	24 ACL versus 24 controls	100% ACLi	x	13 M + 11 F both groups	31.7 ± 9.0 versus 29.1 ± 6.7 (M)	181.9 ± 4.0 versus 182.9 ± 4.9 (M)	84.5 ± 8.3 versus 82.3 ± 10.1 (M)	N/A	EMG	Vastus medialis, vastus lateralis,rectus femoris,biceps femoris, semitendinosus	Weight‐bearing force matching task	x	N/A	Lower relationship between Gluteus medius activity and hip abductor moment in ACLi compared to controls
							25.3 ± 8.2 versus 26.2 ± 7.2 (F)	170.1 ± 5.0 versus 170.2 ± 4.8 (F)	66.7 ± 9.0 versus 64.1 ± 8.7 (F)			Gluteus medius,TFL, adductor muscle group				Greater relationship between adductor muscle group and hip adduction compared to control
Noehren et al. [24]	2014	Cross‐sectional	20 ACL versus 20 controls	100% ACLr	51.5 ± 52 days	100% F	16–40 (21.1 ± 5.9 versus 22.8 ± 3.1)	x	x	BTB, Hamstrings	Dynamometer	Abductors, external rotators	Hip strength, trunk control test and running gait	x	x	No differences in hip strength or frontal and transverse plane kinematics in the ACL group when compared to the healthy control group
Sharifmoradi et al. [31]	2021		8 ACL versus 8 controls	100% ACLr	10 months	x	24.10 ± 1.66	176.8 ± 4.5	76.4 ± 12.5	Hamstrings	Vicon motion analysis system	Gluteus muscles,adductor muscle group, quadriceps femoris,hamstrings	x	x	x	ACLR group had a significant weakness of hip abductor, extensor, and adductor muscles in the affected limb compared with healthy subjects
Nyland et al. [25]	2014	Case series	65 ACL comparing operared side versus controlateral	100% ACLr	5.2 ± 2.9 years	32 M + 33 F	Grp 1: 26.5 versus grp 2: 29.3 versus grp 3: 33.6	grp1: 176.5 versus grp 2: 172.8 versus grp 3: 172.1	grp 1: 76.8 versus grp 2: 76.8 versus grp 3: 79.7	Allograft	EMG	Gluteus maximus, vastus medialis, medial hamstring	Single‐leg hop test	x	Normal active knee extension Week 1, normal quadriceps femoris control during walking Week 2, normal active knee flexion Week 3, 3‐D dynamic neuromuscular stability Week 6, normal functional strength Week 12, normal functional power Week 20, return to sport‐specific training Weeks 20–26	Neuromuscular compensations suggesting a hip bias with increased gluteus maximus and medial hamstring muscle activation was identified at the involved lower extremity among most subjects who perceived high perceived sports capability compared to preinjury status
			Very capable of performing sports activities (Group 1, *n* = 20); capable of performing sports activities (Group 2, *n* = 23), or not capable of performing sports activities (Group 3, *n* = 22).													
Ortiz et al. [26]	2011	Case‐control	13 ACL versus 15 controls	100% ACLr	7.2 ± 4.2 years	100% F	21–35 (25.4± 3.1 versus 24.6 ± 2.6)	167.5 ± 5.9 versus 164.7 ± 6.5	63.2 ± 6.7 versus 58.4 ± 8.9	BTB, Hamstrings, Achilles Allograft	EMG	Gluteus maximus, gluteus medius, quadriceps and hamstrings	Side hopping and crossover hopping tests	x	Initial bracing, neuromuscular re‐education, closed and open kinetic chain strengthening exercises for the hip, knee, and ankle and functional training that included plyometrics and sport drills to enable return to physical activity	No statistically significantl differences between the groups
Srinivasan et al. [36]	2018	Cross‐sectional	70 ACL	47.1% ACLr	17–28 years	21 M + 12 F	45.6 ± 4.5	174.0 ± 9.1	83.0 ± 15.6	x	3‐D motion capture system	Abductors, adductors, internal rotators, external rotators, flexors, extensors	One‐leg hops	Six‐step program: functional exercises with increasing difficulty and functional stability	Knee brace and crutches, physiotherapy improving joint mobility, strength, coordination and balance	Increased variability in lower‐extremity joint couplings has emerged as a conspicuous feature of ACL injured persons in the very long term compared to noninjured controls, independent of treatment
				52.9% ACLi		23 M + 14 F	48.1 ± 5.9	173.5 ± 8.0	87.1 ± 14.9							
			33 controls	N/A		21 M + 12 F	46.7 ± 5.0	176.4 ± 9.8	77.4 ± 14.9							
Rostami et al. [4]	2019	Case‐control	24 ACL versus 12 healthy controls	50% ACLi	18–36 months	100% M	18–30 (23.8 ± 5.5 versus 24.5 ± 2.3 versus 24.9 ± 2.8)	175.3 ± 4.8 versus 174.5 ± 4.6 versus 175 ± 5.2	76.5 ± 5.9 versus 75.3 ± 7.1 versus 74.8 ± 7.5	Patellar tendon, Hamstrings, Allograft	EMG	Gluteus medius and Adductor longus	Single leg vertical drop landing	x	x	ACLr and ACLi participants exhibited decreased peak Gluteus medius activation during single leg vertical drop landing when compared to healthy participants
				50% ACLr												ACLr participants had lower Gluteus medius/Adductor longus co‐activation compared to controls
Dalton et al. [3]	2011	Case‐control	17 ACL versus 17 controls	100% ACLr	3.3 ± 1.7 years	6 M + 11 F both groups	26.8 ± 4.6 versus 25.5 ± 2.9 (M)	176.1 ± 10.6 versus 176.1 ± 5.7 (M)	86.7 ± 15.8 versus 78.6 ± 7.0 (M)	BTB, Hamstrings	EMG and dynamometer	Gluteus medius (EMG)	Star excursion balance test	x	Gym‐based exercise regimens	Greater deficits in hip extensor strength after aerobic exercise in ACLr patients
							23.2 ± 3.4 versus 21.9 ± 1.5 (F)	168.8 ± 7.7 versus 165.7 ± 5.4 (F)	64.7 ± 5.5 versus 62.8 ± 5.9 (F)			Abductors, extensors and external rotators (dynamometer)	Maximum single‐legged vertical jump‐height			No changes in gluteus medius activity after aerobic exercise
Thomas et al. [37]	2013	Case‐control	15 ACL versus 15 controls	100% examined after injury and after reconstruction	68.6 days postinjury	8 M + 7 F versus 7 M + 8 F	20.3 ± 5.4 versus 24.7 ± 3.4	175 ± 10 versus 175 ± 10	74.4 ± 13.3 versus 73.3 ± 13.5	BTB	Dynamometer	Flexors, extensors, abductors, adductors	Concentric strength of each muscle group	Restoration of knee range of motion	Restoration of knee range of motion, flexibility	Adductor strength was greater in participants with ACLr than in controls
					212.5 days after reconstruction										Progressive resistance and closed kinetic chain exercises	No other postoperative strength differences presented between groups
Dingenen et al. [6]	2016		20 ACL versus 20 controls	100% ACLr	23 ± 14 months	5 M + 15 F both groups	22.3 ± 2.3 versus 23.5 ± 2.6	172.2 ± 8.9 versus 171.2 ± 9.4	65.2 ± 8.1 versus 67.6 ± 9.4	x	EMG	Gluteus maximus, gluteus medius, TFL, vastus lateralis, vastus medialis obliquus	Transition from double‐leg to single‐leg stance	x	x	In the eyes open and closed condition, significantly delayed muscle activation onset times of gluteus maximus, gluteus medius, vastus medialis obliquus in ACLr
Dingenen et al. [7]	2015		15 ACL versus 15 controls	100% ACLi	1.4 ± 0.7 months	7 M + 8 F versus 5 M + 10 F	24.7 ± 5.7 versus 24.4 ± 2.1	173.4 ± 8.4 versus 172.2 ± 9.9	69.3 ± 6.2 versus 67.0 ± 10.3	N/A	EMG	Gluteus maximus, gluteus medius, TFL, vastus lateralis, vastus medialis obliquus	Transition from double‐leg to single‐leg stance	x	N/A	In the eyes open condition, significantly delayed muscle activation onset times of gluteus maximus, gluteus medius, vastus lateralis, and vastus medialis obliquus in ACLi
																In the eyes closed condition, significantly delayed muscle activation onset times of tensor fascia latae, vastus lateralis, and vastus medialis obliquus in ACLi
Smeets et al. [32]	2021	Cross‐sectional	20 ACL versus 20 controls	100% ACLr	8.5 ± 1.8 months	14 M + 6 F both groups	23.7 ± 4.3 versus 21.4 ± 1.5	179.1 ± 9.6 versus 179.8 ± 9.7	76.7 ± 11.5 versus 71.5 ± 9.4	Hamstrings	EMG	Gluteus medius, vastus medialis, vastus lateralis, hamstrings medialis, hamstrings lateralis	Stepping‐down task in four different environmental conditions	x	x	Higher hamstrings activation and lower Vastus medialis activation in the injured leg, compared to controls
																Less adjustment in muscle activation levels (smaller increase in Vastus medialis and lateralis activation) in ACLr athletes exposed to platform perturbations compared to controls

*Note*: Summary of the articles included in this study, with the year of publication, study type, number of patients, ACL status, mean date of follow‐up, demographic data, type of ACLr, method of assessment of AMI, test performed, pre‐op and post‐op rehabilitation and key findings.

Abbreviations: ACLi, anterior cruciate ligament injury; ACLr, anterior cruciate ligament reconstruction; AMI, arthrogenic muscle inhibition; BTB, bone‐tendon‐bone; EMG, electromyogram; F, females; M, males; N/A, not applicable; TFL, Tensor Fascia Latae; X, not mentioned in the article.

### Muscles parameters characteristics

The assessment of hip muscles was performed using an electromyogram (EMG) in eight studies, a dynamometer in three studies, and a motion analysis system in two studies.

Four studies evaluated the muscle amplitude, while six studied muscle activation and five reviewed the muscle forces. The results are summarised in Table 3. The results of the muscular forces from the different studies are displayed in Table 4.

**Table 3:** Muscle parameters studied.

	Muscle amplitude	Muscle activation	Muscle force
Flaxman et al. [9]	x		x
Noehren et al. [24]	x		x
Sharifmoradi et al. [31]			x
Nyland et al. [25]	x		
Ortiz et al. [26]	x	x	
Srinivasan et al. [36]		x	
Rostami et al. [4]		x	
Dalton et al. [3]			x
Thomas et al. [37]			x
Dingenen et al. [6]		x	
Dingenen et al. [7]		x	
Smeets et al. [32]		x	

*Note*: Table depicting the three muscle parameters evaluated in each article.

**Table 4:** Forces of hip muscles N/kg (mean ± SD).

	Flaxman et al. [9]	Noehren et al. [24]	Sharifmoradi et al. [31]	Dalton et al. [3]
Gluteus maximus	N/A	N/A	7.5 ± 1.0	N/A
Gluteus medius	N/A	N/A	8.5 ± 1.7	N/A
Gluteus minimus	N/A	N/A	3.8 ± 0.7	N/A
External rotators	0.2 ± 0.1	6.9 ± 4.4	N/A	0.3 ± 0.1 (baseline) versus 0.3 ± 0.1 (after exercise)
Internal rotators	0.2 ± 0.1	N/A	N/A	N/A
Adductors	0.5 ± 0.2	N/A	N/A	N/A
Abductors	0.5 ± 0.1	16.0 ± 4.7	N/A	0.8 ± 0.2 (baseline) versus 0.8 ± 0.3 (after exercise)
Flexors	0.5 ± 0.2	N/A	N/A	N/A
Extensors	0.8 ± 0.2	N/A	N/A	0.5 ± 0.3 (baseline) versus 0.4 ± 0.2 (after exercise)
Rectus	N/A	N/A	22.2 ± 5.4	N/A
Vastus medialis	N/A	N/A	11.7 ± 2.3	N/A
Vastus lateralis	N/A	N/A	36.9 ± 5.8	N/A
Biceps femoris	N/A	N/A	3.5 ± 0.7	N/A
Semitendinosus	N/A	N/A	4.6 ± 0.4	N/A
Semimembranous	N/A	N/A	14.0 ± 2.0	N/A
Adductor brevis	N/A	N/A	3.5 ± 0.7	N/A
Adductor longus	N/A	N/A	7.9 ± 1.9	N/A
Adductor magnus	N/A	N/A	2.7 ± 0.8	N/A
Iliacus	N/A	N/A	13.4 ± 2.8	N/A
Psoas	N/A	N/A	17.0 ± 2.1	N/A

*Note*: Force of every single muscle in the hip according to every article.

Abbreviation: N/A, not applicable.

### Best evidence synthesis

The current best evidence suggests that AMI of the gluteus medius may occur following ACL injury and reconstruction. Direct evidence is provided by two studies (Flaxman et al. [9] Sharifmoradi et al. [31]) that underline reduced gluteus medius activation or hip abductor weakness using EMG and motion analysis. These alterations are consistent with neural inhibition potentially driven by joint damage, swelling, or altered proprioception. On the other hand, other studies (e.g., Noehren et al. [24]; Ortiz et al. [3]) failed to show significant differences in gluteus medius strength or activation compared to controls. This was attributed to variability in individual response as well as potential recovery through rehabilitation. Neuromuscular compensations at the hip after ACL reconstruction were suggested by Nyland et al. [25], who indirectly substantiated impaired hip muscle function. Given the methodological heterogeneity (EMG, dynamometry, functional tests) and differences in participant characteristics, the evidence remains mixed.

## DISCUSSION

This systematic review shed the light on the neuromuscular alterations regarding the Gluteus medius in a context of ACL pathology, whether the patient was ACL deficient, or underwent an ACL reconstruction. Since the available studies are limited and lack high level of evidence, it is difficult to clearly affirm our hypothesis. There seems to be an arthrogenic inhibition of the Gluteus medius of patients with ACL injury, regardless of the conservative or surgical management of the ACLi. It is important to distinguish between the effects of the initial ACLi and those following ACLr. While the injury itself may trigger immediate neuromuscular inhibition of the Gluteus medius through altered afferent input and protective mechanisms, surgical reconstruction introduces additional factors such as graft harvest, pain, swelling and altered kinematics that may further influence muscle inhibition.

Among the 12 articles included in this study, two showed a direct correlation between the force of the Gluteus medius and the reconstruction of the ACL [9, 31], while three others reported a diminished activity onset of the Gluteus medius in patients with injured and/or reconstructed ACL [4, 6, 7].

While Sharifmoradi et al. found a significant weakness of hip abductors, extensors and adductors in the affected limb of eight ACLr patients compared with healthy subjects, using a motion analysis system [31], Flaxman et al. revealed a lower activity of the Gluteus medius as well as the hip abductor moment in ACL injured patients using a reliable isometric weight‐bearing force matching protocol [9].

On the other hand, a delayed activation onset time of the Gluteus medius while performing a single leg vertical drop landing [5] or when transitioning from double‐leg to single‐leg stance [6, 7] results in less resistance to hip adduction and internal rotation. This pattern is linked to a higher re‐injury risk since it reproduces the mechanism of noncontact ACL injuries [4, 24].

Every single article of this systematic review compared a cohort of ACLi and/or ACLr patients to a control group, except one single paper by Nyland et al. [25] The assessment of the Gluteus medius function and force in the healthy population was completely normal, which highlights the possible implication of the pathologic ACL in the AMI of the Gluteus medius.

On the other hand, this systematic review showed inconclusive results on abductor forces. The variability in the assessment of muscle function around the hip has given way to some contradicting results. In fact, Noehren et al. have not found any difference in hip strength in the frontal and transversal planes of individuals with ACLr when compared to a healthy control group [24]. However, trunk control differed significantly during running following completion of the ACLr rehabilitation program.

Similarly, Dalton et al. only found a significant reduction of force in the extensors muscle group of ACLr patients after aerobic exercise, but not for isometric hip external rotation or abduction strength [3]. This was due to the relatively low demand that was placed on muscles like Gluteus medius during forward walking. This test might not be the optimal one to challenge the frontal‐plane hip stabilisers in order to expose any differences between groups postexercise. Nevertheless, individuals with ACLr exhibited reduced Gluteus medius EMG activation during the anterior reach of the Star Excursion Balance Test (a single‐legged dynamic balance while the participant accomplishes a maximal reach task with the contralateral limb), when compared to the control group. This emphasises the crucial role that the performed tests play in uncovering muscle weaknesses. Thomas et al., on the other hand, used a dynamometer to examine the concentric strength of each muscle group in ACLr patients [37]. They were unable to identify a hip‐abductor weakness and suggested that it was related to the postoperative rehabilitation which strengthened the muscle groups.

The existence of AMI of the hip muscles has yet to be established. All in all, it is difficult to conclude if the Gluteus medius's force directly diminishes after ACL injury or reconstruction. However, abnormal adaptation of hip stabilising muscles is repeatedly seen in almost all these studied articles, suggesting a functional neuromuscular alteration. These abnormal movements may originate from poor muscular recruitment or activation [4]. Although ACLr restores mechanical stability, it does not necessarily equates to the recovery of normal neuromuscular control [6, 25].

These altered neuromuscular patterns observed in ACLi/ACLr may reflect an arthrogenic muscle response (AMR), a reflexive and protective mechanism involving the inhibition and/or facilitation of specific muscles surrounding the affected joint [11, 30, 32, 33, 34, 35]. This serves to prevent any potential harmful movement by modifying neuromuscular control. In the ACLi context, the typical response results in Quadriceps inhibition (ACL antagonist) and increased excitability of the Hamstrings (ACL agonists), thus providing a compensatory mechanism to stabilise the joint. This imbalance hampers the ability to activate motor units effectively, which limits strength development and ultimately hinders recovery and rehabilitation [3, 9, 12].

Up‐and‐coming evidence suggests that neuromuscular impairment due to ACLi extends across the kinetic chain and isn't just confined to the injured joint [37]. This is due to interconnected neural pathways and central nervous system (CNS) adaptations, like the ones shown in animal models between the rectus femoris and the sartorius, thereby explaining how weakness in the rectus femoris could potentially affect hip abductors as well [16].

In AMR, the brain's ability to perceive joint position and execute appropriate motor responses is compromised, which explains postural control deficits like impaired single‐leg balance with eyes open or closed in patients with ACLr [3]. The reason for this is twofold. Firstly, the mechanoreceptors of the injured knee are disrupted (due to joint effusion, inflammation, pain, ligament laxity or injury). With no sensory feedback, the ability to achieve the suitable motor response is altered. Therefore, the patient develops a compensatory gait pattern with a distorted muscle activation strategy during functional tasks or athletic activities, all of which increase the susceptibility to re‐injury [3, 7, 13]. Second, as a result of the altered proprioceptive afferent input, the brain undergoes neuroplastic changes, with a higher frontal cortex activation and a decreased parietal activation [6, 32]. This phenomenon has been well‐documented with EEG revealing increased frontal theta power and decreased parietal alpha‐2 power in patients with ACLr during proprioceptive tasks [7]. These CNS adaptations are not merely compensatory but represent a fundamental reorganisation of the sensorimotor program. This is true even after surgery and ACLr, which indicates that mechanical restoration does not fully resolve the neurosensory deficit [6, 7, 25]. ACLi should therefore be perceived not only as a mechanical disruption but also as a de‐afferentation injury that triggers local and central neuromuscular consequences. This neuroplasticity significantly impacts functional performance even after the structural integrity of the ligament has been restored [25].

In this systematic review, four studies identified a delayed activation onset time of the Gluteus medius [4, 6, 7, 36]. These may not solely reflect postinjury adaptations, but could also represent pre‐existing neuromuscular deficits that predisposed the patient to an ACLi in the first place [29, 32]. This could explain the elevated risk of bilateral ACL re‐injury due to persistent impairments in neuromuscular control throughout the kinetic chain [40]. When transitioning from a double‐leg to a single‐leg stance in 20 patients who underwent ACLr, Dingenen et al. found no significant differences in muscle activation onset times between the operated and nonoperated legs [6]. This suggests that neuromuscular control deficits are not confined to the reconstructed limb, showcasing instead the influence of CNS adaptations following ACLr.

With a single‐leg hop test on 65 patients who underwent an ACLr, Nyland et al. was able to identify neuromuscular compensations at the hip, suggesting an abnormal adaptation of the hip stabilising muscles [25]. By increasing neuromuscular activation of the Gluteus maximus and medial hamstring muscles in the involved limb, individuals may rely on compensatory muscle activation strategies to optimise performance.

In his case‐control study, Ortiz et al. didn't find any statistical difference between 13 ACLr and 15 controls during side hopping and crossover hopping tests when it came to Gluteus medius activation [26]. However, during side‐hopping manoeuvre, they manifested an increased valgus moment at the hip. Since the study assessed other muscle groups, it was possible to also determine that this category of patients had higher hip flexion and hip adduction angles, which could potentially reveal a certain degree of ‘weakness’ of the abductor muscles. Even if women with ACLr were able to compensate the neuromuscular stability of their knee joint, it is evident that a certain neuromuscular adaptation is occurring around the hip. This was affirmed by Srinivasan et al., who highlighted the following pattern: ACLi individuals exhibit increased lower extremity joint coordination variability during functional tasks, independently of the time elapsed since surgery, or the treatment modality (reconstruction versus physical therapy) [36]. Ultimately, this underlines a long‐term neuromuscular control alteration after ACLi.

The 12 studies required various methods of evaluation of the hip muscles. Electromyography records muscle electrical activity during contractions, thus revealing activation patterns and neuromuscular control mechanisms [8, 18, 25, 27, 28, 29, 30, 31]. Dynamometers can be handheld or isokinetic devices. They quantify muscle strength and torque [3, 24, 37]. As for the 3D motion analysis, it employs infrared cameras and reflective markers to capture precise 3D kinematic data, facilitating in‐depth movement analysis as well as range of motion of articulations [31, 36]. If these methods are combined, a complete evaluation of the musculoskeletal function in both clinical and experimental settings can be achieved. This variability in muscle study makes a certain standardisation of data more difficult. This might explain certain conflicting results that were exposed.

Future research should explore whether targeted hip muscle training can reduce persistent neuromuscular deficits after ACL injury or reconstruction. It has been shown that the hip plays a pivotal role in conferring knee stability by coordination the movement between the trunk and the knee [24]. Should the hip be dysfunctional for any reason, altered knee loading ensues and consequently a higher risk of re‐injury [10, 20, 22, 42]. Therefore, the hip asymmetry observed between normal and injured lower limbs has the potential to increase the risk of re‐injury of the ACL [24, 31, 36]. This asymmetry in muscle forces can persist after ACLr even despite thorough postoperative rehabilitation. Understanding that diminished Gluteus medius activity after ACLr or ACLi is a neuromuscular adaptation is pivotal in order to better incorporate strategical postinjury and postoperative rehabilitation to better activate this muscle and enhance lower extremity stability during activity [3, 4, 31].

When transitioning from double‐leg to single‐leg stance, visual input is removed, movement speed is altered, and the level of movement readiness is changed. This constitutes a valuable task for probing the sensorimotor system as CNS should proactively stabilise the lower extremity in response to the imminent postural change. Relying on this principle, Dingenen et al. were able to underline that even after a full return to sport and successful rehabilitation, ACLr patients continued to exhibit delayed hip muscle activation onset times [6]. Since injury disrupts afferent sensory input, central adaptations in sensorimotor control persists even after mechanical restoration through surgery. Thus, targeting the underlying neurophysiological changes should be the new paradigm in the CNS rehabilitation strategy instead of solely focusing on peripheral muscle function. This could be critical in optimising long‐term recovery and reducing re‐injury risk [7, 25, 32]. It has been already proven that AMI of the quadriceps after an ACLi is easily reversible with some targeted exercises [34, 35]. The same principles should be therefore applied for AMI of the Gluteus medius. No article in this systematic review mentioned a clear rehabilitation program that would specifically target the weakened Gluteus medius. However, many papers attributed this weakness to a nonspecific rehabilitation program. They insist on a more personalised rehabilitation targeting the neurological pathway to counter the AMI of the Gluteus medius [6, 7, 25, 31, 36, 37]. This can optimise hip function post‐ACL injury.

This review has several limitations. First, the number of studies directly addressing gluteus medius inhibition after ACL injury remains limited, which restricts the strength of the conclusions. Second, heterogeneity in study designs, assessment tools (EMG protocols, dynamometry and motion analysis), and patient populations (injury chronicity, surgical technique, rehabilitation status) makes direct comparison challenging. Third, most included studies were of small sample size and did not consistently control for confounding variables such as preinjury hip strength or concomitant injuries. Moreover, this review may be influenced by publication bias, as studies with nonsignificant findings are less likely to be published. Limiting the review to English language could have also excluded relevant research published in other languages. Finally, the absence of longitudinal studies prevents establishing causality or the temporal evolution of AMI in the gluteus medius. Larger, prospective longitudinal studies should clarify the onset, progression and persistence of Gluteus medius inhibition after ACL injury and reconstruction by standardising protocols and biomechanical assessments.

## CONCLUSION

Gluteus medius dysfunction may occur in some individuals following ACL injury or reconstruction and can be indicative of AMI as part of a more complex somatosensory disrupted pathway. This can compromise hip stability and hinders functional outcome and recovery. The reason behind the arousal of AMI of the Gluteus medius in a certain category of ACL patients remains to be determined. Future research should investigate whether targeted hip muscle training can mitigate persistent neuromuscular deficits following ACL injury.

## AUTHOR CONTRIBUTIONS

*Writing original draft and editing*: Jean Tarchichi. *Data curation and formal analysis*: Jad Zalaket. *Writing review*: Nicolas Graveleau and Pierre Laboudie. *Conceptualisation and writing review and editing*: Nicolas Bouguennec.

## CONFLICT OF INTEREST STATEMENT

The authors declare no conflicts of interest.

## ETHICS STATEMENT

The authors have nothing to report.

## Supporting information

Appendix 1: Best evidence synthesis.

## Data Availability

The authors have nothing to report.
