# Supplementary material for: Evidence for arthrogenic inhibition of the gluteus medius after anterior cruciate ligament injury: A systematic review
Source: J Exp Orthop. 2025 Nov 14;12(4):e70551. doi: 10.1002/jeo2.70551 (PMC12616644; doi:10.1002/jeo2.70551)
Supplement: Supplementary file 1 — Appendix 1: Best evidence synthesis. [file JEO2-12-e70551-s001.docx]

| Strong evidence | Provided by consistent, statistically significant findings in outcome measures in at least two high-quality RCTs |
| --- | --- |
| Moderate evidence | Provided by consistent, statistically significant findings in least one low quality RCT or high-quality CCS |
| Limited evidence | Provided by consistent, statistically significant findings in outcome measures in at least one high-quality RCT or provided by consistent, statistically significant findings in outcome measures in at least two high-quality CCS (in the absence of high-quality RCTs) |
| Indicative findings | Provided by consistent, statistically significant findings in outcome and/or process measures in at least one high-quality CCS or low-quality RCT (in the absence of high-quality RCTs) or provided by consistent, statistically significant findings in outcome and/or process measures in at least two noncontrolled studies with sufficient quality (in the absence of RCTs and CCS) |
| No or insufficient evidence | In the case that results of eligible studies do not meet the criteria for one of the above stated levels of evidence or in the case of conflicting (statistical significant positive and statistical significant negative) results among RCTs and CCS or in the case of no eligible studies  If the number of studies that show evidence is <0% of the total number of  studies found within the same category of methodological quality and study design (RCT, CCS, or other design), no evidence will be stated |

Appendix 1: Best evidence synthesis

Levels of evidence proposed by Van Tulder et al. [39] and adapted by Steultjens et al. [36]
